# Angiolipoma of the chest wall: a case report

**DOI:** 10.1186/s40792-022-01384-y

**Published:** 2022-02-21

**Authors:** Takahiro Omori, Sho Nakamura

**Affiliations:** Department of Thoracic Surgery, Yokosuka General Hospital Uwamachi, 2-36 Uwamachi, Yokosuka, Kanagawa 238-8567 Japan

**Keywords:** Angiolipoma, Chest wall, Video-assisted thoracic surgery (VATS)

## Abstract

**Background:**

Angiolipoma is a rare histological variant of lipoma. Angiolipoma commonly occurs in the subcutaneous tissues of the extremity and trunk. There are few reports of angiolipoma occurring in the chest wall.

**Case presentation:**

A 78-year-old woman was referred to our hospital for evaluation of angina pectoris. Coronary computed tomography (CT) showed a soft tissue nodule in the left chest wall by chance. Enhanced chest CT showed a heterogeneous enhanced nodule in the left chest wall. On magnetic resonance imaging (MRI), the lesion showed low signal intensity on T1-weighted images, heterogeneous high signal intensity on T2-weighted images and high signal intensity on fat-suppressed T2-weighted images. The lesion showed heterogeneous enhanced effect on gadolinium-based contrast agent. These radiological findings suggested neurogenic tumor with abundant blood flow or hemangioma. Video-assisted thoracic surgery (VATS) was performed for both diagnostic and therapeutic purposes. Histopathological examination of the tumor showed mature adipose tissue and capillary hyperplasia containing fibrin thrombi. These appearances were consistent with angiolipoma. She had an uneventful recovery and did not show recurrence until 8 months post-surgery.

**Conclusions:**

Angiolipoma of the chest wall is extremely rare. Preoperative diagnosis is very difficult because the imaging findings of angiolipoma vary depending on the amount of vascular component and fat component, so surgical resection is suggested to be both diagnostic and therapeutic.

## Background

Angiolipoma is a rare histological variant of lipoma with a vascular component and commonly occurs in the subcutaneous tissues of the extremities and trunk [1, 2]. Angiolipoma of the chest wall is extremely rare, and, to our best knowledge, only six cases of angiolipoma of the chest wall have been reported [3–8].

## Case presentation

**Fig. 1:**
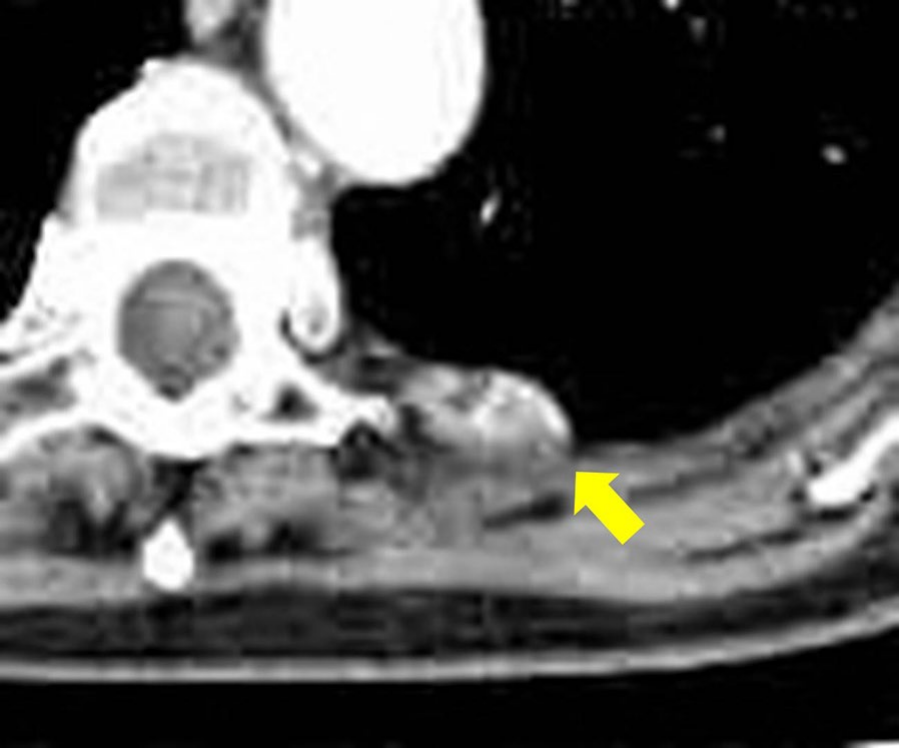
Enhanced chest CT scan findings. Enhanced chest CT showed an about 22-mm-sized heterogeneous enhanced nodule in the left chest wall at the fifth–sixth intercostal level

**Fig. 2:**
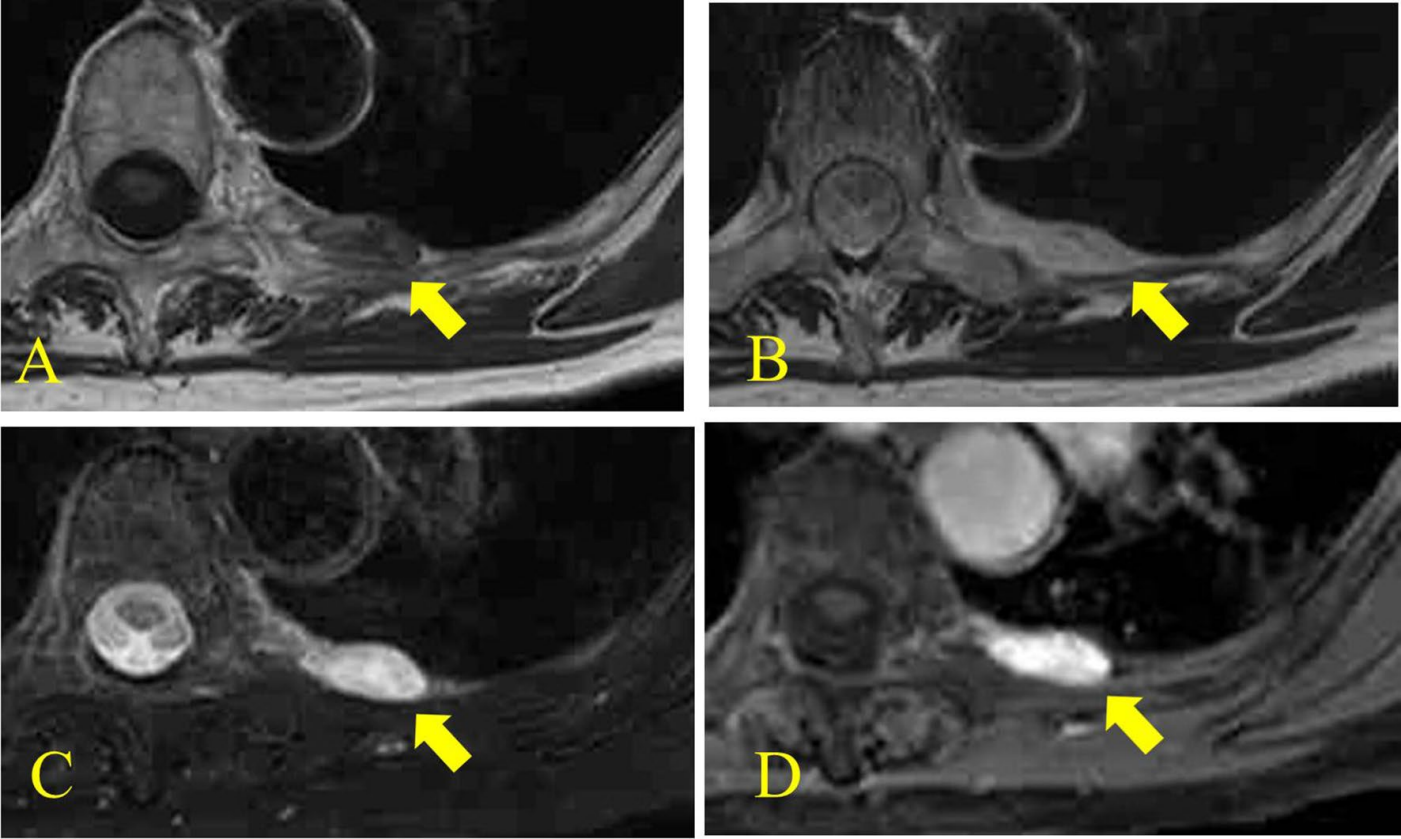
Chest MRI findings. The lesion showed low signal intensity on T1-weighted images (**A**), heterogeneous high signal intensity on T2-weighted images (**B**) and high signal intensity on fat-suppressed T2-weighted images (**C**). The lesion showed heterogeneous enhanced effect on gadolinium-based contrast agent (**D**)

A 78-year-old woman was referred to our hospital for evaluation of angina pectoris. Coronary computed tomography (CT) showed no significant findings in the coronary arteries, but showed a soft tissue nodule in the left chest wall by chance. Enhanced chest CT showed an about 22-mm-sized heterogeneous enhanced nodule in the left chest wall at the fifth–sixth intercostal level (Fig. 1). On magnetic resonance imaging (MRI), the lesion showed low signal intensity on T1-weighted images (Fig. 2A), heterogeneous high signal intensity on T2-weighted images (Fig. 2B) and high signal intensity on fat-suppressed T2-weighted images (Fig. 2C). The lesion showed heterogeneous enhanced effect on gadolinium-based contrast agent (Fig. 2D).

**Fig. 3:**
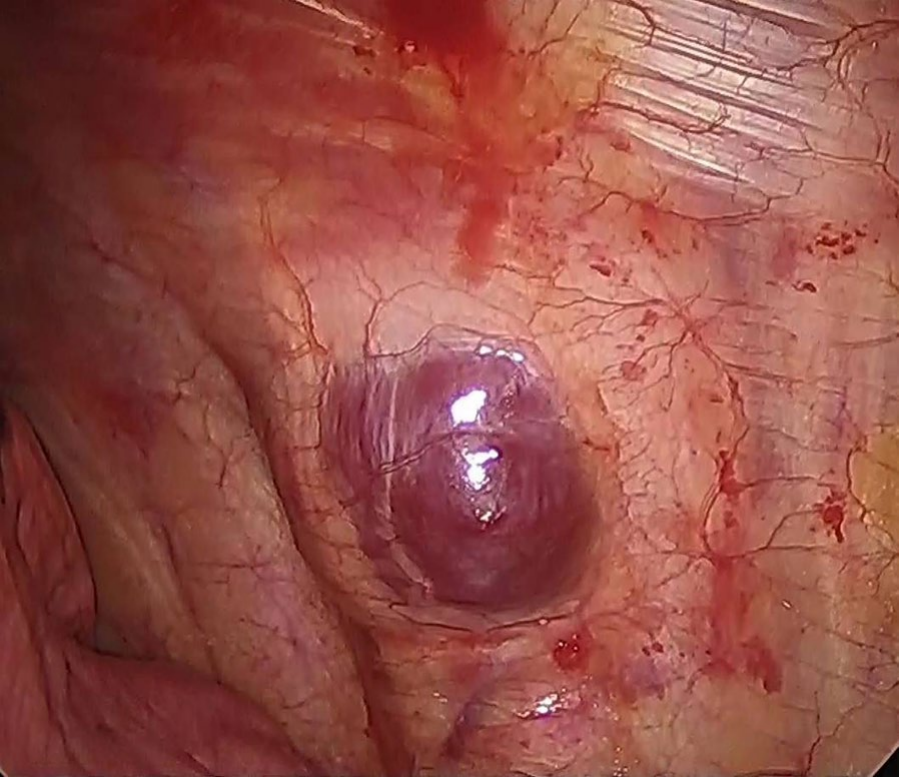
Intraoperative findings. A dark red, soft tumor was found in the left chest wall

**Fig. 4:**
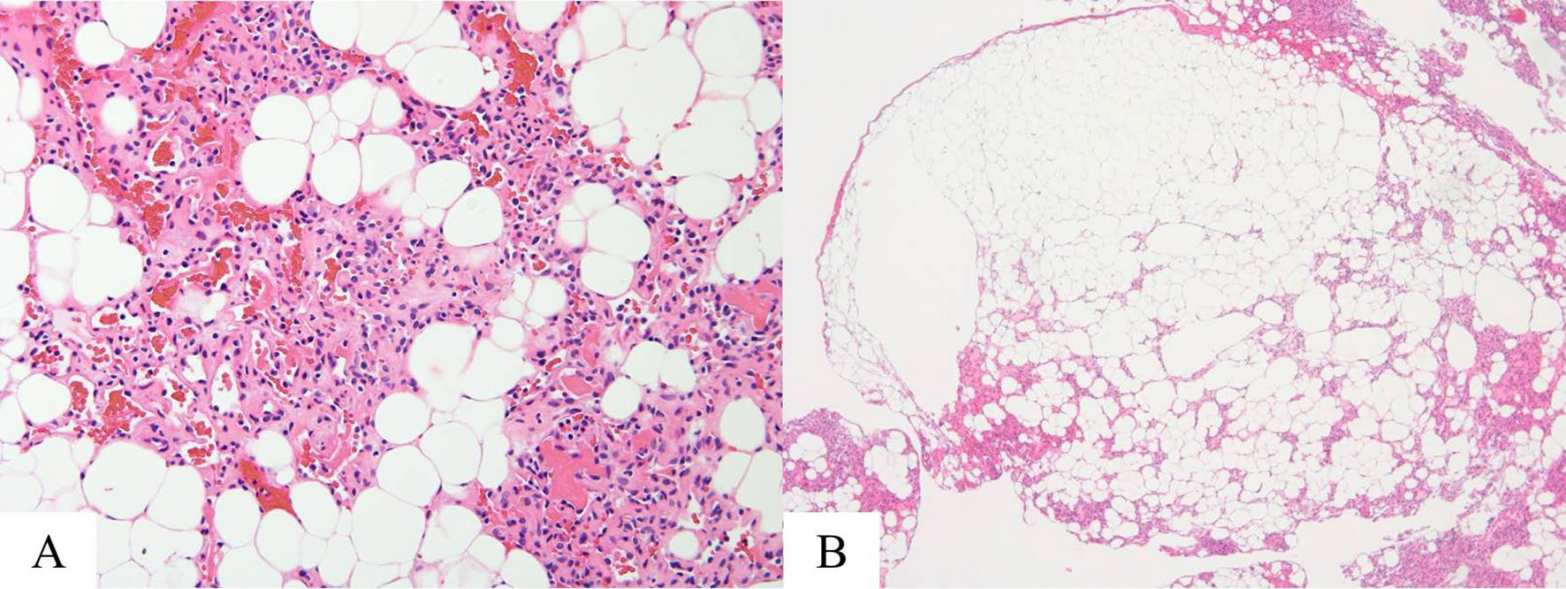
Histopathological findings. Histopathological examination of the tumor showed mature adipose tissue and capillary hyperplasia containing fibrin thrombi (**A** H&E, ×200). The tumor was well-defined and encapsulated (**B** H&E, ×40)

These radiological findings suggested neurogenic tumor with abundant blood flow or hemangioma. For diagnostic and therapeutic purposes, a four-port complete video-assisted thoracic surgery (VATS) was performed. One 2-cm port access incision was made in the fifth intercostal middle-axillary line, and three 7 mm of them were made. A dark red, soft tumor was found in the left chest wall (Fig. 3). The tumor was bleeding easily. The tumor was encapsulated and showed no invasion into the surrounding tissues, therefore local excision was performed. Histopathological examination of the tumor showed mature adipose tissue and capillary hyperplasia containing fibrin thrombi (Fig. 4A). The tumor was well-defined and encapsulated (Fig. 4B). These appearances were consistent with non-infiltrating angiolipoma. The postoperative course was uneventful, and the patient was discharged on the 5th day after the operation. She did not show recurrence until 8 months post-surgery.

## Discussion

Angiolipoma is a rare histological variant of lipoma with a vascular component. Angiolipoma was first reported by Bowen in 1912 [9] and was established angiolipoma as an entity by Howard and Helwig in 1960 [1]. Angiolipoma commonly occurs in the subcutaneous tissues of the extremities and trunk, and the incidence of angiolipoma is 5–17% of all the lipomas [1, 2]. The patient ages range from 16 to 58 years, with a median age of 24 and they are male predominant [1]. Angiolipoma of the chest wall is extremely rare, and, to our best knowledge, only six cases of angiolipoma of the chest wall have been reported [3–8].

Angiolipoma has morphological features that consist of mature adipose tissue with angiomatous proliferation. Blood vessels in angiolipoma often contains fibrin thrombi, which is the characteristic of angiolipoma [1, 10]. Angiolipoma is classified as two histologic types: infiltrating and non-infiltrating [2]. Infiltrating angiolipoma is noncapsulated tumor and infiltrates surrounding tissue. Non-infiltrating angiolipoma is encapsulated, which is the most common form.

Preoperative diagnosis of angiolipoma of the chest wall is challenging because the imaging findings of angiolipoma vary depending on the amount of fat component and vascular component. Findings of MRI showed homogeneous high signal intensity on both T1- and T2-weighted images with decreased signal on fat-suppressed images which reflects a fat component, on the other hand heterogeneous high signal intensity on only T2-weighted images which reflects a vascular component [7, 11, 12]. In our case, MRI of the lesion showed low signal intensity on T1-weighted images and high signal intensity on fat-suppressed images. We suggested this case was poorly fat component and vascular component-rich angiolipoma.

Treatment of angiolipoma is complete surgical excision for both infiltrating and non-infiltrating types. Infiltrating angiolipoma frequently infiltrates surrounding tissue, thus wide excision is recommended, and in some cases, chest wall resection with ribs are also required [2, 7]. Non-infiltrating angiolipoma has been treated by local excision [2]. Infiltrating angiolipoma has a recurrence rate of 50%, however a good prognosis can be expected with radiotherapy and additional excision. On the other hand, non-infiltrating angiolipoma shows no tendency to recurrence [2, 12, 13]. In our case, the tumor was encapsulated and showed no infiltration into the surrounding tissues, which was a non-infiltrating angiolipoma, and local excision was performed.

## Conclusions

In conclusion, we described our experience with a case of angiolipoma of the chest wall. Angiolipoma of the chest wall is extremely rare. Preoperative diagnosis is very difficult and surgical resection is suggested to be both diagnostic and therapeutic.

## Data Availability

The data are not available for public access because of patient privacy concerns.

## Abbreviations

CT: Computed tomography
MRI: Magnetic resonance imaging
VATS: Video-assisted thoracic surgery

## References

[1] Howard WR, Helwig EB (1960). Angiolipoma. Arch Dermatol.

[2] Lin JJ, Lin F (1974). Two entities in angiolipoma: a study of 459 cases of lipoma with review of literature on infiltrating angiolipoma. Cancer.

[3] Biondetti PR, Fiore D, Perin B, Ravasini R (1982). Infiltrative angiolipoma of the thoracoabdominal wall. J Comput Assist Tomogr.

[4] Deviri E, Levinsky L, Shaklai M, Lavie G, Levy MJ (1987). Total excision of a giant angiolipoma of chest wall with A-V malformation and with the use of an autotransfusion system. J Cardiovasc Surg.

[5] Mayooran N, Tarazi M, O’Brien O, Hinchion J (2016). Infiltrating angiolipoma of the chest wall: a rare clinical entity. J Surg Case Rep.

[6] Komatsu T, Takahashi K, Fujinaga T (2013). Chest wall angiolipoma complicating von Recklinghausen disease. Ann Thorac Surg.

[7] Hamano A, Suzuki K, Saito T, Kuwatsuru R, Oh S, Suzuki K (2013). Infiltrating angiolipoma of the thoracic wall: a case report. Open J Clin Diagn.

[8] Sakamoto R, Tanaka T, Murakami J, Nakamura T, Yoshimine S, Hamano K (2019). A case of non-infiltrating angiolipoma of the chest wall mimicking radiological infiltration of the rib. Jpn J Chest Surg.

[9] Bowen JT (1912). Multiple subcutaneous hemangiomas, together with multiple lipomas, occurring in enormous numbers in an otherwise healthy, muscular subject. Am J Med Sci.

[10] Dixon AY, McGregor DH, Lee SH (1981). Angiolipomas: an ultrastructural and clinicopathological study. Hum Pathol.

[11] Matsuoka Y, Kurose K, Nakagawa O, Katsuyama J (1988). Magnetic resonance imaging of infiltrating angiolipoma of the neck. Surg Neurol.

[12] Arenaz BJ, Luaces R, Lorenzo FF, Garcia-Rozado A, Crespo EJL, Fonseca CE, Lopez-Cedrun JL (2010). Angiolipoma in head and neck: report of two cases and review of the literature. Int J Oral Maxillofac Surg.

[13] Gonzalez-Crussi F, Enneking WF, Arean VM (1966). Infiltrating angiolipoma. J Bone Joint Surg.

